# Emotion Dysregulation and Inflammation in African-American Women with Type 2 Diabetes

**DOI:** 10.1155/2016/8926840

**Published:** 2016-07-17

**Authors:** Abigail Powers, Vasiliki Michopoulos, Karen Conneely, Rachel Gluck, Hayley Dixon, Joseph Wilson, Tanja Jovanovic, Thaddeus W. W. Pace, Guillermo E. Umpierrez, Kerry J. Ressler, Bekh Bradley, Charles F. Gillespie

**Affiliations:** ^1^Department of Psychiatry and Behavioral Sciences, Emory University School of Medicine, Jesse Hill Jr. Drive, Atlanta, GA 30306, USA; ^2^Department of Human Genetics, Emory University School of Medicine, Atlanta, GA, USA; ^3^College of Nursing & College of Medicine (Psychiatry), University of Arizona, Tucson, AZ, USA; ^4^Division of Endocrinology, Department of Medicine, Emory University School of Medicine, Atlanta, GA, USA; ^5^Center for Depression, Anxiety, and Stress Research, Harvard University, Cambridge, MA, USA; ^6^Atlanta VA Medical Center, Atlanta, GA, USA

## Abstract

C-reactive protein (CRP), a marker of systemic inflammation, has been associated with major depressive disorder (MDD) and posttraumatic stress disorder (PTSD). Emotion dysregulation is a transdiagnostic risk factor for many psychological disorders associated with chronic inflammatory state. The objective of this study was to determine whether inflammation is associated with emotion dysregulation in women with type 2 diabetes mellitus (T2DM). We examined associations between trauma exposure, MDD, PTSD, emotion dysregulation, and CRP among 40 African-American women with T2DM recruited from an urban hospital. Emotion dysregulation was measured using the Difficulties in Emotion Regulation Scale. PTSD and MDD were measured with structured clinical interviews. Child abuse and lifetime trauma load were also assessed. Analyses showed that both emotion dysregulation and current MDD were significantly associated with higher levels of CRP (*p* < 0.01). Current PTSD was not significantly related to CRP. In a regression model, emotion dysregulation was significantly associated with higher CRP (*p* < 0.001) independent of body mass index, trauma exposure, and MDD diagnosis. These findings suggest that emotion dysregulation may be an important risk factor for chronic inflammation beyond already known risk factors among women with T2DM, though a causal relationship cannot be determined from this study.

## 1. Introduction

Major depressive disorder (MDD) and posttraumatic stress disorder (PTSD) have been associated with higher incidence of cardiovascular disease [1–4] and diabetes [5, 6]. One mechanism that may contribute to the comorbidity between psychopathology and adverse health outcomes is chronic inflammation, whose role in atherosclerosis is well established [7, 8]. C-reactive protein (CRP) is a marker of systemic inflammation and provides a useful indicator of chronic inflammation. Increased circulating concentrations of CRP are associated with MDD [7, 9–11]. Increased circulating concentrations of CRP have also been described in PTSD [12–14]; however, the relationship is not as clear as other data are equivocal [15–17]. Because inflammation may be a key factor in understanding the significant comorbidity between psychiatric disorders and physical diseases, establishing other psychological factors that help explain the relationship between inflammation and psychiatric disorders and how problems with inflammation may develop is critical.

Ample evidence indicates that early life stress is associated with chronic inflammation in adulthood [18, 19], and this may be particularly true among African Americans [20]. For example, a recent meta-analysis on the role of childhood maltreatment (i.e., childhood sexual, physical, and emotional abuse and physical and emotional neglect) in inflammation in adulthood demonstrated a significant positive association between exposure to childhood trauma and higher circulating concentrations of CRP [21]. Childhood maltreatment exposure may be particularly detrimental to the immune system via problematic alterations in the function of the hypothalamic pituitary adrenal (HPA) axis, a major component of the body's stress response system and a system that both modulates and is modulated by inflammatory processes [22, 23]. Based on this evidence, some posit that chronic inflammation may be one mediator in the relationship between early trauma exposure and the later development of psychiatric disorders [21]. It is important to note that the results of the meta-analysis on childhood trauma and inflammation suggested a relatively subtle (although significant) effect of trauma exposure on inflammatory activation suggesting that there may be other important components to this pathway that should be considered.

Emotion dysregulation, or deficits in awareness and management of intense negative emotional states, is a transdiagnostic risk factor for the development and maintenance of many psychological disorders, including depression and PTSD [23, 24], and may be another psychological factor that impacts chronic inflammation. Importantly, emotional development begins early in life and developmental research has shown a strong relationship between exposure to childhood maltreatment and the development of emotion regulation deficits in adolescence and adulthood [25–27]. This may occur in part because emotion regulation strategies are learned by caregivers and when family environments are harmful or unsupportive, children are less likely to be exposed to appropriate regulatory behavior and more likely to experience emotional invalidation where emotional expression is ignored, rejected, or punished [28]. Therefore, emotion dysregulation may be an important mechanism by which early life adversity confers lifetime risk for psychological disorders [25, 26]. Based on the strong link between childhood maltreatment and emotion dysregulation, it is very possible that emotion dysregulation may also relate to chronic inflammation.

Recent research in fact suggests that emotion functioning is related to inflammation. A longitudinal study examining emotional functioning in 7-year-old children showed that inappropriate self-regulation and distress proneness during childhood predicted higher circulating concentrations of CRP in adulthood [29]. Other cross-sectional research on adults looked specifically at emotion regulation and showed that reappraisal (an adaptive emotion regulation skill) was associated with lower circulating concentrations of CRP while suppression (a maladaptive emotion regulation skill) was associated with elevated circulating concentrations of CRP [30]. Taken together, these studies indicate that emotion dysregulation could be important in predicting chronic inflammation, although no studies to our knowledge have examined the specific association between emotion dysregulation and circulating concentrations of CRP among traumatized adults with high rates of depression and PTSD.

One population at particular risk for elevated systemic inflammation is individuals with type 2 diabetes mellitus (T2DM). Multiple recent longitudinal studies show that elevated circulating concentrations of CRP put individuals at greater risk of developing T2DM [31, 32]. Understanding psychological factors that may be associated with increased inflammation levels in individuals with T2DM could be useful when considering how to improve treatments. Therefore, the goal of the present study was to examine the differential associations between trauma exposure, emotion dysregulation, current MDD, current PTSD, and CRP concentrations in a highly traumatized urban, minority sample of women with T2DM. We hypothesized that emotion dysregulation would be associated with higher concentrations of CRP independent of the effects of trauma exposure and current psychiatric diagnoses (i.e., PTSD and MDD).

## 2. Material and Methods

### 2.1. Procedure

Participants were drawn from astudy of risk factors for the development of PTSD in a low socioeconomic, urban minority population. Participants were recruited from waiting rooms in the diabetic, gynecology, and primary care medical clinics at a publically funded hospital in Atlanta, Georgia. We did not narrow recruitment to specific criteria but approachedany individual in the waiting room. To be eligible for participation, subjects had to be between the ages of 18 and 65 and able to give informed consent (see [33] for full details regarding study procedures). The investigation was carried out in accordance with the latest version of the Declaration of Helsinki and informed consent of the participants was obtained after the nature of the procedures had been fully explained. After signing the informed consent approved by the Emory Institutional Review Board and the Research Oversight Committee of Grady Memorial Hospital, an interview was administered with questionnaires regarding trauma history and psychological variables. Trained research assistants administered this interview (approximately 45–75 minutes).

A subgroup of female participants with T2DM was chosen for a separate associated study. These women returned to participate in structured clinical interviews and phlebotomy. Exclusion criteria included current bipolar or psychotic disorder diagnosis, alcohol or substance dependence, treatment for an autoimmune disorder, treatment with nonsteroidal anti-inflammatory, glucocorticoid, or anticonvulsant, and current treatment with an antipsychotic, benzodiazepine, or antidepressant. On the morning of the interview, height and weight were measured for calculation of body mass index (BMI) and fasting blood samples were collected for later batch assessment of CRP concentrations (approximately 2 weeks after initial assessment).

### 2.2. Participants

The current study included 40 diabetic African-American women with a mean age of 51.88 years (SD = 7.57 years, range = 32–65). Education levels were as follows: 18.8% reported less than high school education, 21.9% reported having a high school diploma or GED, 37.5% reported having some college or technical school education, and 21.9% reported graduating from technical school or college. Only 25.0% of participants were employed and 84.4% had a household monthly income of $1999 or less.

### 2.3. Measures

#### 2.3.1. Difficulties in Emotion Regulation Scale (DERS)

The DERS is a 36-item psychometrically validated [34] self-report measure of emotion regulation difficulties. It measures several aspects of emotion regulation, including awareness and understanding of one's emotions, acceptance of negative emotions, the ability to successfully engage in goal-directed behavior and control impulsive behavior when experiencing negative emotions, and the ability to use situationally appropriate emotion regulation strategies. For the present study the overall scale, as well as six subscales, of emotion regulation was examined. The internal consistency of the DERS total scale was high (*α* = 0.92).

#### 2.3.2. Traumatic Events Inventory (TEI)

The TEI is a 14-item screening instrument for lifetime history of traumatic events. It was administered to detail frequency and type of trauma(s) experienced; consistent with prior research [33], total level of trauma exposure was measured by a sum score reflecting the total frequency of different types of trauma (e.g., car accident, sexual assault) a participant had been exposed to over the course of their life. For this study, the TEI was used to measure frequency of lifetime trauma exposure. The number of traumatic experiences reflects the total number of different types of events that an individual experienced or witnessed. In order to separate lifetime trauma exposure and childhood abuse, we excluded the childhood abuse items from the TEI (i.e., exposure to sexual abuse, physical abuse, and emotional abuse).

#### 2.3.3. Childhood Trauma Questionnaire (CTQ)

The CTQ [35] is a 25-item, reliable, and valid self-report instrument assessing sexual, physical, emotional abuse, and neglect in childhood (*α* = 0.92 in current study). Bernstein and Fink [36] established scores for none, mild, moderate, and severe for each type of abuse. For descriptive purposes, the data from the CTQ were used to create a categorical variable to account for the presence or absence of moderate-to-severe reported exposure to emotional (score ≥ 13), physical (score ≥ 10), and sexual (score ≥ 8) abuse in childhood (0 = none or mild abuse; 1 = the presence of moderate or severe abuse scores for at least one of the three types of abuse). A continuous measure of overall severity of childhood abuse exposure was also calculated and used as the measure of child abuse severity in all analyses.

#### 2.3.4. Clinician-Administered PTSD Scale (CAPS)

The CAPS is an interviewer-administered psychometrically validated diagnostic instrument measuring PTSD [37, 38]. It includes items that rate social and occupational functioning, global PTSD symptom severity, and the validity of participant's responses. The CAPS assesses current PTSD and was used to determine presence/absence of a PTSD diagnosis.

#### 2.3.5. MINI International Neuropsychiatric Interview (MINI)

MINI [39] is a structured diagnostic interview that assesses mood, anxiety, substance use, and psychotic disorders based on DSM-IV-TR criteria. MINI has shown good reliability and validity across different samples [39, 40]. For the present study, only the current major depression section was used to assess presence/absence of current MDD.

#### 2.3.6. Body Mass Index (BMI)

Body mass index is calculated as BMI = body mass (kg)/(height (m))^2^. Mean BMI for this sample was 36.53 (SD = 7.30, range = 21–53.80).

#### 2.3.7. Highly Sensitive CRP

Serum samples were stored at −80°C until the time of highly sensitive CRP (hsCRP) assay. Serum hsCRP concentrations were determined using an immunoturbidimetric assay from Sekisui Diagnostics (Lexington, MA) on the Beckman AU480 chemistry analyzer, with an interassay coefficient of variation (CV) of 5.2% and an intra-assay CV of 3.1%. Individuals with circulating concentrations of hsCRP >20 mg/L were excluded from analysis because hsCRP > 20 mg/L suggests the presence of an active infection or other illnesses that could seriously confound study findings (two individuals were excluded from analyses (hsCRP = 26.25 and 61.69)). Circulating concentrations of hsCRP averaged 5.70 mg/L (SD = 4.81, range = 0.23–18.43).

#### 2.3.8. Glucose

Plasma samples of glucose were stored at −80°C until the time of assay. Glucose is measured by enzymatic methods on the Beckman AU480 using reagents from Beckman Coulter (Fullerton, CA). Average glucose level for this sample was 125.05 (SD = 38.31, range = 35.70–208.15).

#### 2.3.9. Hemoglobin A1c (HbA1c)

Whole blood samples were stored at −80°C until the time of assay. HbA1c was measured using high performance liquid chromatography by ARUP laboratories (Salt Lake City, Utah). Average HbA1c for this sample was 7.61 (SD = 1.71, range = 4.90–13.60).

### 2.4. Data Analysis

The overall analytic approach was to examine the predictive utility of trauma, current PTSD, current MDD, and emotion dysregulation on hsCRP concentrations. We first examined the distributions of all key predictor variables. hsCRP concentrations, trauma exposure, and emotion dysregulation variables were positively skewed. However, the level of skewness (range: −0.32–1.96) in this sample fell within acceptable parameters for the sample size on all variables except child abuse severity [41] and the difficulty with emotion regulation strategies dimension of emotion dysregulation. Each had one outlier which affected the level of kurtosis, but results remained the same with or without these outliers included and so they remained in the models presented. Descriptive statistics of the variables of interest were computed (Table 1). Differences in trauma level, emotion dysregulation, and BMI were also examined by both MDD and PTSD diagnoses using analysis of variance (Table 1). For variables of interest, bivariate correlations (continuous variables) and a univariate analysis of variance (categorical variables) were also computed to determine associations with hsCRP in this sample. Then, based on the results of the correlational analyses, a series of linear regression models was fit to examine the unique predictive value of child abuse, current MDD, and emotion dysregulation on hsCRP. Potential covariates for regression analysis were first identified based on previous research suggesting associations with circulating concentrations of hsCRP: age, income, BMI, hemoglobin A1C, and baseline blood glucose level [9, 42–45]. Due to the small sample size and risk for low power in detecting significant effects, associations between hsCRP and these potential covariates were first assessed to determine if their inclusion in the regression model was warranted. No significant differences emerged between any of the variables and hsCRP except for BMI (*r* = 0.40, *p* = 0.01) and therefore only BMI was included as a covariate in the regression analysis. All analyses were conducted with* SPSS 23.0 *software package (twelve primary analyses were conducted resulting in a Bonferroni correction value of *p* < 0.004).

## 3. Results

This was a highly traumatized sample, with all participants reporting the experience of at least one type of trauma in their lifetime (excluding child abuse; M = 4.31, SD = 2.28). Many participants were also exposed to childhood abuse (i.e. sexual, physical, or emotional abuse), with 35.0% of the sample reporting exposure to moderate-to-severe child abuse. Rates of depression and PTSD were also high in this sample, with 32.5% (*n* = 13) meeting criteria for current MDD and 32.5% (*n* = 13) meeting criteria for current PTSD. The majority of those individuals that met diagnostic criteria for at least one diagnosis in fact met diagnostic criteria for both MDD and PTSD (22.5% of overall sample, *n* = 9). As shown in Table 1, individuals with depression and PTSD were more likely to report higher levels of child abuse (*p* < 0.01 and *p* < 0.001, resp.) and emotion dysregulation (*p* < 0.01 and *p* < 0.05, resp.). Rates of other trauma exposure and BMI were not significantly different across groups.

To determine the extent of association between trauma exposure, emotion dysregulation, and hsCRP, we first calculated Pearson correlation coefficients among our variables of interest (see Table 2). BMI was also included in these analyses to evaluate independent associations with the variables of interest. Overall emotion dysregulation was significantly positively correlated with hsCRP concentrations (*p* < 0.001). When looking at the six dimensions of emotion dysregulation, all six were significantly positively correlated with difficulty controlling impulses and lack of emotion regulation strategies showing the strongest correlations (*p* < 0.001). All significant associations except for lack of awareness of emotions would survive correction for multiple comparisons. BMI (*p* = 0.01) was also positively correlated with hsCRP. The association between child abuse severity and hsCRP trended toward significance (*p* = 0.067). However, overall trauma exposure (excluding child abuse) was not significantly correlated with hsCRP. Regarding associations between trauma exposure and emotion dysregulation, child abuse severity was associated with overall emotion dysregulation (*p* < 0.05) as well as nonacceptance of emotions (*p* = 0.01) and difficulty with goal-directed behavior in the presence of strong emotions (*p* = 0.05). Overall trauma exposure (excluding child abuse) was not associated with emotion dysregulation. However, child abuse severity and overall trauma exposure (excluding abuse) were significantly correlated with each other (*p* < 0.05). Regarding BMI, only one emotion dysregulation dimension—lack of awareness of emotions—was significantly associated with BMI (*p* < 0.05). Neither trauma exposure variable was significantly associated with BMI.

**Figure 1:**
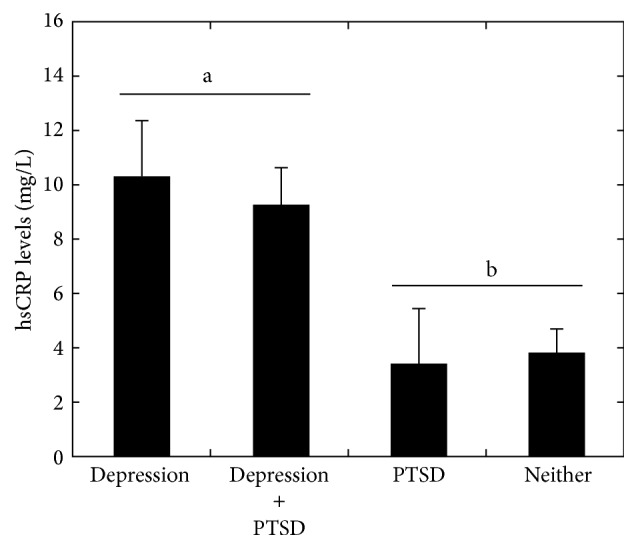
Univariate analysis of variance results predicting mean hsCRP levels by current depression and PTSD diagnoses. Significant main effect of depression denoted by letters (*p* < 0.001).

Univariate analysis of variance was then used to examine mean differences in hsCRP by PTSD and MDD diagnosis. As shown in Figure 1, univariate analysis of variance results showed that there was a significant increase in mean hsCRP across current MDD diagnosis (*F* = 19.12, *p* < 0.001) but not current PTSD diagnosis (*F* = 2.87, *p* = 0.10). There was no significant interaction between MDD and PTSD diagnoses.

Next, a series of linear regression models was run to test the differential associations of child abuse, current MDD, and emotion dysregulation with hsCRP. BMI was entered in the first step of the regression model as a covariate. Then, child abuse severity was entered into the second step of the regression model. Next, current MDD was entered into the third step of the regression model. Finally, emotion dysregulation was entered into the fourth step of the regression model. As shown in Table 3, when BMI was included in step 1, higher BMI was significantly related to higher hsCRP levels (*p* < 0.05). When child abuse severity was included in step 2, it was not significant in predicting hsCRP. In step 3, when current MDD was entered into the regression model, MDD was significantly predictive of hsCRP (*p* < 0.01), accounting for 13% of the variance in hsCRP independent of BMI and child abuse severity. When overall emotion dysregulation was included in the model in step 4, emotion dysregulation was significantly predictive of hsCRP (*p* < 0.001) above and beyond BMI, child abuse severity, and current MDD, surviving correction for multiple comparisons and accounting for 25% of unique variance in predicting higher hsCRP concentrations. Including PTSD in the model did not change the results; a model was also run with continuous measures of depression (MINI) and PTSD (CAPS) and significant results did not change.

## 4. Discussion

To our knowledge, the current study is the first to evaluate the differential relationship between emotion dysregulation, trauma exposure, psychiatric disorders, and circulating CRP concentrations in a traumatized sample of women. In support of our hypothesis, within this sample of African-American females with T2DM, we found that emotion dysregulation was significantly associated with higher peripheral concentrations of CRP. This result is consistent with recent research which has shown that emotional functioning in childhood predicts elevated CRP in adulthood [29] and expands previous findings to demonstrate that emotion dysregulation was significantly predictive of elevated CRP above and beyond already known risk factors, including BMI, trauma, and current MDD.

Although all dimensions of emotion dysregulation measured were significantly related to higher CRP, difficulty engaging in goal-directed behavior in the presence of strong emotions and a lack of strategies for managing strong negative emotions showed the strongest associations with elevated concentrations of CRP. These results indicate that heightened inflammation in those with high emotion dysregulation in the current study may be due to chronic activation of neuroendocrine and immune systems in response to chronic stress exposure as a result of a lack of effective strategies for managing negative emotions. Indeed, exposure to repeating and unrelenting psychosocial stressors results in the dysregulation of the feedback mechanisms that regulate the activity of the HPA axis and the inflammatory system [21, 22]. Under normal conditions, glucocorticoids are released in response to stressors that act to inhibit the activity of the immune system [46]. However, overactivation of the HPA axis in response to chronic stress exposure leads to dysfunction of the HPA axis and glucocorticoid responsiveness that leads to an attenuation of glucocorticoid-induced inhibition of the inflammatory response [46]. Thus, it is possible that an inability to manage negative emotions in response to psychosocial stressor exposure with adaptive strategies facilitates increased inflammation. Although the direction of causality cannot be established from an association study, improving emotion regulation strategies and individuals' ability to tolerate strong emotions and not act on them may be a useful area to focus on in treatment with individuals who have comorbid psychopathology and chronic inflammation. Future studies with a controlled experimental design are needed to assess whether improvements in emotional regulation can lead to reductions in inflammation. It is also critical that more research be conducted to dissect the associations between the various dimensions of emotion dysregulation and chronic inflammation to inform treatment decisions.

Consistent with previous research, we also found that current depression was associated with a proinflammatory state. Indeed, individuals with MDD have been shown in meta-analyses to have elevated CRP [7, 9–11], as well as increased levels of interleukin-6 (IL-6) and tumor necrosis factor-*α* (TNF-*α*) [9, 47]. Causal pathways between depression and inflammation have been debated and much of the evidence has focused on the role of proinflammatory cytokines in the development of depressive symptoms and “sickness behavior” (e.g., anhedonia, sleep, and appetite changes [48–51]). However, not all individuals with depression show a heightened inflammatory state, and it is likely that in fact the positive association between depression and inflammation is a result of a complex and bidirectional process in which components of the central nervous system (e.g., HPA axis, autonomic nervous system) alter inflammation and depression which in turn impact one another [9]. Our finding that current depression is associated with higher CRP in females is helpful since some recent population-based studies found sex differences in associations between depression and CRP, such that the relationship was only found for men [10, 11]. However, other studies looking specifically at women have found strong associations between MDD and higher levels of CRP in women [52, 53], and our finding indicates further support for this relationship among women. It will be important to continue evaluating sex differences in the association between inflammation and psychopathology as the type of population studied clearly impacts the interpretation and generalizability of the association.

While depression was significantly associated with CRP, current PTSD was not, which contradicts some previous findings [12–14]. However, the literature on this association is mixed, with studies also showing a negative association between PTSD and CRP [15] or no association at all [16, 17]. It is likely that the specificity of our sample (highly traumatized African-American females with T2DM) contributed to these results and therefore limits the generalizability of these results to other populations. There may be other pathways associated with the stress response system and emotion dysregulation that may be more relevant to PTSD than inflammation, such as enhanced glucocorticoid negative feedback of the HPA axis and heightened activity of the sympathetic nervous system [54]. Ongoing studies in our lab are beginning to examine how immune, neuroendocrine, and autonomic function all relate to PTSD and MDD in the context of acute stress.

Overall trauma exposure was also not related to circulating concentrations of CRP. It is possible that this is in part due to the fact that all women included in the study had been exposed to traumatic events (mean = 4.23 types of events). However, although not significant in this sample, we did see a trend toward a positive association between child abuse and peripheral concentrations of CRP. Our small sample size made us underpowered to detect small effects, and it is likely that with more women included, this effect would be statistically significant. This result would fit with previous research finding a relationship between child abuse exposure and inflammation in adulthood [55]. Of note, emotion dysregulation and child abuse were significantly related, and based on the strong effect found between emotion dysregulation and CRP, it is possible that emotion dysregulation is the mechanism by which early trauma contributes to chronic inflammation, although this could not be explored in this cross-sectional study. More research is needed to determine potential risk pathways of trauma exposure and emotion regulation problems on the development of inflammation and should be examined in both traumatized and nontraumatized populations.

The current study has some limitations that should be kept in mind when interpreting the results. First, the study was cross-sectional in nature, which does not allow for the determination of causality. It is certainly possible that chronic inflammation leads to higher levels of emotion dysregulation, as some research has shown that inflammation can lead to mood and emotional changes [56]. However, the strong evidence that emotion dysregulation develops throughout childhood and is impacted by early life trauma [25–27, 57–59], along with initial evidence that the emotional functioning in childhood predicts adult CRP concentrations [29], provides support for emotion dysregulation as an underlying mechanism that might lead to chronic inflammation and later health problems such as cardiovascular disease and diabetes. In addition, emotion dysregulation was measured using a self-report questionnaire (DERS). This means that participants needed to have insight into their emotion regulation difficulties in order to report them. A lack of insight could have underestimated the severity of emotion dysregulation within this group of women. However, the DERS has shown good construct validity in its strong relationship with psychiatric symptoms and outcomes (e.g., PTSD symptoms, self-harm behavior [34, 60–62]). Recent research has also shown associations between the poor impulse control dimension of the DERS and lower activation of the rostral anterior cingulate cortex during an impulse control task, suggesting that this self-report measure of emotion dysregulation is related to already known neural mechanisms associated with adaptive emotion regulation, thus further supporting its validity. Another limitation is that the homogeneity of the study population means that the results may not be generalizable to other samples. This sample included all African-American women with T2DM and high levels of trauma exposure, thus exceeding the rates of psychiatric disorders generally found in community samples [33]. However, the homogeneous sample is a strength as African-American women with T2DM are clearly an at-risk sample that would benefit from improved interventions. Past research has shown increased inflammatory biomarkers in low-socioeconomic-status African-American communities [63, 64] which is consistent with our sample of traumatized T2DM women, as overall peripheral concentrations of CRP in this sample were very high (mean = 5.70 mg/L, SD = 4.80). Finally, the small sample size of this study means that we may not have had enough power to detect small, yet clinically important associations between the variables examined. This was evident in the association between child abuse severity and higher concentrations of CRP (*r* = 0.29, *p* = 0.067) that was trending toward significance. This is the first study to examine the complex relationships between trauma, psychiatric disorders, self-reported emotion dysregulation, and CRP and therefore more research with larger sample sizes is needed to replicate these findings.

## 5. Conclusions

Within at-risk groups it may be particularly beneficial to target psychological treatment interventions that may also then impact inflammation. CRP is easily measured and is a significant predictor of risk for serious physical conditions like cardiovascular disease. Future research should examine how psychological interventions for depression and/or PTSD might differentially affect emotion dysregulation and CRP concentrations and whether changes in one might then influence the status of the other. There is already some evidence that reductions in chronic inflammation can result from treating depression [65] and that reducing inflammation decreases affective symptoms [66]. Emotion dysregulation may be an even more useful treatment target since it cuts across psychiatric disorders and can be implemented in the context of various treatment modalities (e.g., cognitive-behavioral, acceptance and commitment, and dialectical behavior therapies) and may be more easily integrated into nonpsychiatric settings (e.g., primary care and diabetes clinics) where brief skills training is more feasible than long-term psychotherapy.

## Figures and Tables

**Table 1:** Descriptive characteristics of variables of interest.

	Overall sample	Current depression		Current PTSD	
		0	1	0	1
	*N* = 40	*N* = 27	*N* = 13	*N* = 27	*N* = 13
	Mean (SD, range)	Mean (SD)	Mean (SD)	Mean (SD)	Mean (SD)
Overall trauma load (excluding child abuse)	4.31 (2.27, 1–12)	4.52 (2.38)	3.86 (2.02)	3.97 (2.00)	5.00 (2.70)
Child abuse severity	37.81 (16.11, 25–100)	33.48 (10.59)	46.81 (21.68)^*∗∗*^	31.37 (7.65)	51.19 (20.71)^*∗∗∗*^
Emotion dysregulation total	72.75 (24.81, 39–145)	64.78 (19.56)	89.31 (27.06)^*∗∗*^	67.22 (22.26)	84.23 (26.76)^*∗*^
*Nonacceptance of emotions*	12.10 (5.48, 6–27)	10.67 (4.69)	15.08 (5.98)^*∗*^	11.26 (5.35)	13.85 (5.54)
*Difficulty with goal-directed behavior*	11.52 (5.00, 5–25)	9.48 (2.89)	15.77 (5.88)^*∗∗∗*^	9.93 (3.11)	14.85 (6.54)^*∗∗*^
*Difficulty controlling impulses*	11.20 (4.865, 6–21)	9.48 (2.89)	13.31 (4.92)	10.52 (5.09)	12.62 (4.17)
*Lack of awareness of emotions*	13.40 (4.868, 6–21)	12.70 (4.18)	14.85 (4.43)	12.93 (4.46)	14.38 (4.01)
*Lack of emotion regulation strategies*	14.48 (5.99, 8–36)	12.63 (4.60)	18.31 (6.86)^*∗∗*^	13.11 (4.99)	17.31 (7.05)^*∗*^
*Lack of clarity of emotional experience*	10.05 (4.47, 5–23)	9.11 (4.44)	12.00 (4.02)	9.48 (4.45)	11.23 (4.46)
Body mass index	36.53 (7.30, 21–53.80)	35.15 (6.65)	39.35 (7.16)	36.17 (7.31)	37.30 (7.52)

Differences between groups were tested using one-way analysis of variance:  ^*∗*^ *p* ≤ 0.05; ^*∗∗*^ *p* ≤ 0.01; and ^*∗∗∗*^ *p* ≤ 0.001.

Note: trauma load was measured using TEI; child abuse severity was measured using CTQ; emotion dysregulation was measured using DERS; current PTSD was measured using CAPS; and current depressive episode was measured using MINI.

**Table 2:** Bivariate Pearson's correlations (*r*) between variables of interest.

	1	2	3	4	5	6	7	8	9	10	11
Emotion dysregulation total	—										
*Nonacceptance of emotions*	0.82^*∗∗∗*^	—									
*Difficulty with goal-directed behavior*	0.82^*∗∗∗*^	0.56^*∗∗∗*^	—								
*Difficulty controlling impulses*	0.80^*∗∗∗*^	0.58^*∗∗∗*^	0.58^*∗∗∗*^	—							
*Lack of awareness of emotions*	0.65^*∗∗∗*^	0.33^*∗*^	0.47^*∗∗∗*^	0.44^*∗∗*^	—						
*Lack of emotion regulation strategies*	0.93^*∗∗∗*^	0.76^*∗∗∗*^	0.74^*∗∗∗*^	0.65^*∗∗∗*^	0.55^*∗∗∗*^	—					
*Lack of clarity of emotional experience*	0.89^*∗∗∗*^	0.69^*∗∗∗*^	0.65^*∗∗∗*^	0.68^*∗∗∗*^	0.51^*∗∗∗*^	0.83^*∗∗∗*^	—				
Other trauma exposure	−0.02	−0.01	0.07	0.14	−0.11	−0.04	−0.18	—			
Child abuse severity	0.32^*∗*^	0.40^*∗∗*^	0.31^*∗*^	0.20	0.15	0.23	0.27	0.38^*∗*^	—		
hsCRP	0.73^*∗∗∗*^	0.60^*∗∗∗*^	0.67^*∗∗∗*^	0.55^*∗∗∗*^	0.44^*∗∗*^	0.73^*∗∗∗*^	0.56^*∗∗∗*^	0.14	0.29^†^	—	
Body mass index	0.21	0.14	0.14	0.06	0.32^*∗*^	0.24	0.14	−0.04	0.13	0.40^*∗∗*^	—

^*∗*^ *p* ≤ 0.05; ^*∗∗*^ *p* ≤ 0.01; ^*∗∗∗*^ *p* ≤ 0.001; and ^†^ *p* = 0.067.

Note: emotion dysregulation was measured using DERS, child abuse severity was measured using CTQ, and trauma exposure was measured using TEI.

**Table 3:** Linear regression model predicting current hsCRP levels from trauma exposure, current MDD diagnosis, and overall emotion dysregulation symptoms.

	*β*	*t*	*p*	*R*	*R* ^2^ change	*F* change	*p* change
Step 1				0.42	0.17	5.69	0.02^*∗*^
BMI	0.42	2.39	0.02^*∗*^				

Step 2				0.49	0.07	2.34	0.14
BMI	0.40	2.31	0.03^*∗*^				
Child abuse severity	0.26	1.53	0.14				

Step 3				0.61	0.13	5.23	0.03^*∗*^
BMI	0.28	1.71	0.10				
Child abuse severity	0.08	0.47	0.64				
Current MDD	0.42	2.29	0.03^*∗*^				

Step 4				0.79	0.25	16.39	<0.001
BMI	0.22	1.68	0.11				
Child abuse severity	−0.05	−0.32	0.75				
Current MDD	0.24	1.59	0.13				
Emotion dysregulation	0.58	4.05	<0.001				

^*∗*^ *p* ≤ 0.05.

Note: child abuse severity was measured using CTQ; emotion dysregulation was measured using DERS; and current depressive episode was measured using MINI.
